# Correlation between Serum Fatty Acid Binding Protein 4 (FABP4) Levels and Cardiac Function in Patients with Thalassemia Major

**DOI:** 10.1155/2021/5130628

**Published:** 2021-12-27

**Authors:** Pandji I. Fianza, Anita Rahmawati, Shofura Afifah, Suhendra Praptama, Mohammad Ghozali, Teddy A. Sihite, Djatnika Setiabudi, Mas R. A. A. Syamsunarno, Suthat Fucharoen, Ramdan Panigoro

**Affiliations:** ^1^Department of Internal Medicine, Division of Hematology and Medical Oncology, Faculty of Medicine, Universitas Padjadjaran/Hasan Sadikin General Hospital, Bandung, West Java, Indonesia; ^2^Research Center of Medical Genetics, Faculty of Medicine, Universitas Padjadjaran, Bandung, West Java, Indonesia; ^3^Doctoral Study Program, Faculty of Medicine, Universitas Padjadjaran, Bandung, West Java, Indonesia; ^4^Department of Biomedical Sciences, Faculty of Medicine, Universitas Padjadjaran, Bandung, West Java, Indonesia; ^5^Department of Cardiology and Vascular Medicine, Faculty of Medicine, Universitas Padjadjaran/Hasan Sadikin General Hospital, Bandung, West Java, Indonesia; ^6^Department of Child Health, Faculty of Medicine, Universitas Padjadjaran/Hasan Sadikin General Hospital, Bandung, West Java, Indonesia; ^7^Thalassemia Research Center, Institute of Molecular Biosciences, Mahidol University, Nakhonpathom, Thailand

## Abstract

**Background:**

Patients with thalassemia major may suffer from complications due to iron overload. It has been suggested that several adipokines may play a potential role in the development of complications in thalassemia. Fatty acid-binding protein 4 (FABP4) is one of the adipokines, bridging several aspects of metabolic and inflammatory pathways. Little is known about the relationship between this adipokine and cardiac and liver function, especially in patients with thalassemia major.

**Aims:**

This study is aimed at determining serum FABP4 levels in patients with thalassemia major and whether its concentration correlated with serum ferritin levels, as well as cardiac and liver function.

**Methods:**

Thalassemia major outpatients (*n* = 48) completed laboratory examination, echocardiography, and electrocardiography.

**Results:**

The mean age was 21.9 ± 8.0 years. A negative and weak correlation between serum ferritin and FABP4 was observed (*r* = −0.291, *p* < 0.05). In addition, there was moderate and positive correlation between left atrial volume index (LAVI) and FABP4 (*r* = 0.316, *p* < 0.05).

**Conclusions:**

Serum FABP4 correlated with serum ferritin and cardiac function in patients with thalassemia major. FABP4 may be a potential clinical biomarker for cardiac dysfunction via metabolic and inflammatory pathways due to iron accumulation and toxicity in patients with thalassemia major.

## 1. Introduction

Thalassemia is one of the most common hereditary diseases that interfere with the synthesis of the normal globin chain, occurring in no less than 2.4 out of every 1000 live births, globally [1–3]. Ineffective erythropoiesis and hemolysis encompass the main reason of anemia in thalassemia [3, 4]. Severe anemia from early life is characteristic of thalassemia major thus requiring routine blood transfusion to survive [4]. However, a continuous load of iron due to repeating transfusion will result in iron overload because the body has no active elimination pathway for iron [5, 6]. Therefore, to reduce iron accumulation and its related complications, iron chelation therapy is needed [7, 8].

The report suggests that in thalassemia, the liver is the primary target organ dysfunction, augmented by initiation of free radical reactions due to iron overload-induced oxidative stress [9, 10]. Delayed in diagnosis and treatment contributed to development of liver iron overload into cirrhosis [11]. On the other hand, among complications of iron overload, 63.6-71% of morbidity and mortality were affected by cardiomyopathy [12, 13]. Cardiac arrhythmias and congestive heart failure have been recognized as the most common cause of death in thalassemia [8, 12, 14–16].

Iron is one of the essential minerals for cellular function; however, iron overload, as happened in thalassemia patients, may result in cell injury [12, 17]. Iron could initiate free radical reaction and potentially impair cellular metabolism, including carbohydrates, proteins, lipids, and nucleic acids [5, 17, 18]. A chronic inflammatory condition existed in patients with thalassemia major [19–21]. Inflammatory state resulted from reactive oxygen species production, lipid peroxidation, and other metabolic changes due to iron toxicity are risk factors for cardiac dysfunctions, such as heart failure and left ventricular dysfunction [22].

Fatty acid-binding proteins (FABPs) are a group of proteins that adjust intracellular lipid transport [23]. FABPs have molecular weights of 14-15 kDa and have high-affinity bonding with hydrophobic ligands, including unsaturated and saturated fatty acids [24]. Among FABPs, fatty acid-binding protein 4 (FABP4), known as adipocyte FABP, is one of the adipokines that act as a novel biomarker for the metabolic and inflammatory state [25], and it may be affected in patients with thalassemia major because of the iron excess. FABP4 is mostly expressed in adipocytes and macrophages and is associated with several aspects of the metabolic and inflammatory pathway, such as metabolic syndrome and cardiovascular disease [26]. Elevation of FABP4 levels may play a role in left ventricular dysfunction [22]. It is also associated with metabolic syndrome, type 2 diabetes, nonalcoholic steatohepatitis, atherogenic dyslipidemia, and heart failure [27].

It has been reported that several adipokines may have a potential role in the pathophysiological mechanism of complications in thalassemia [19–21, 28, 29]. However, the study about association of FABP4 and cardiac function in patients with thalassemia major is still limited. Therefore, the objectives of this study were to evaluate the serum FABP4 levels in patients with thalassemia major and whether its concentration was affected by iron excess assessed by serum ferritin levels. Given the increasing evidence of iron toxicity-induced inflammation-related metabolic and cardiovascular disturbances, the relationships between FABP4 and function of the target organ, including heart and liver were also investigated.

## 2. Materials and Methods

### 2.1. Subjects

The study subjects were the family members of the Thalassemia Parents Association in Bandung, West Java, Indonesia. Between June and October 2017, an explanation and invitation were sent to patients with thalassemia major who regularly visited the outpatient clinics at the Department of Internal Medicine, Division of Hematology and Medical Oncology, Hasan Sadikin General Hospital, Bandung. Consecutive agreements from 48 subjects were received. Patients, who were older than 14 years old and have received transfusion for at least 2 years, were included in the study. Patients on acute and/or severe infection were excluded by history taking and physical examination to prevent interference of the results of serum ferritin [30]. This research study was undertaken with the understanding and written informed consent of each patient and with the approval of the Ethics Committee of Faculty of Medicine, Universitas Padjadjaran, Bandung (approval number: 662/UN6.C10/PN/2017). This study conformed to the principles outlined in the Declaration of Helsinki.

Diagnosis of thalassemia was based on the clinical history and laboratory confirmation. Patients' data were retrieved from medical records. Upon obtaining written informed consent, age and sex were recorded. Additionally, the detailed variables regarding age at the start of transfusion and the interval of blood transfusion were also obtained based on patients' information. All patients had received chelating drugs, including deferasirox (20 mg/kg), deferiprone (75 mg/kg), or deferoxamine (30-50 mg/kg) either as monotherapy or combination. All subjects underwent blood and cardiac examination, including electrocardiography (ECG) and echocardiography.

### 2.2. Measurement of Laboratory Parameters

Before transfusion, after overnight fasting, in the morning, blood was collected through venipuncture from each patient and centrifuged for 15 minutes at 3,000 rpm to separate the serum. Blood parameters, including pretransfusion hemoglobin, were measured using an automated hematology analyzer (Sysmex XN-®Series™ Hematology Analyzers). Serum ferritin level was assessed by the electrochemiluminescence immunoassay (ECLIA) method using ADVIA Centaur XPT Immunoassay System Siemens Healthineers. Although the specificity of serum ferritin is sometimes problematic for the estimation of body iron [5], it may expect siderosis when it is >2500 ng/mL [13, 30]. The concentration of liver enzymes, including aspartate transaminase (AST) and alanine transaminase (ALT), was determined using Automated Hematology Analyzer Dimension® EXL™ 200. The serum concentration of FABP4 was quantified using a commercially available enzyme-linked immunosorbent (ELISA) assay kit for FABP4 (CSB-E12995h; Cusabio, Wuhan, China). Briefly, the assay employed the competitive inhibition enzyme immunoassay technique. The microtiter plates provided in the kit had been precoated with FABP4. The assay was conducted according to the manufacturer's instructions. A calibration curve was constructed by plotting the absorbance values at 450 nm vs. the FABP4 concentrations of the calibrators, and concentrations of unknown samples were determined by use of this calibration curve. In our study, the lower detection limit (sensitivity) for FABP4 was 15.6 pg/mL, and the detection range was 62.5-4000 pg/mL. The intra- and interassay coefficient variances in the kit were <8% and <10%, respectively.

### 2.3. Assessment of the Cardiovascular Function

One week after blood transfusion, the cardiovascular function was assessed. Echocardiography was performed by a well-experienced echocardiographer who was blinded to the clinical data, using Vivid S6 equipped with a 1.5–3.6 MHz frequency transducer. Standard two-dimensional and tissue Doppler echocardiography parameters were determined according to the American Society of Echocardiography [31]. Left ventricular end-systolic volume (LVESV) was assessed from apical four-chamber view. Left ventricular ejection fraction (LVEF), fractional shortening (FS), left ventricular mass, and mass index were measured to see left ventricular function. A normal amount of LVEF was considered >55%, while >30% for normal LVFS. Velocities in early (*E*) and late (*A*) diastole were recorded. In the age group of 16-40 years old, which included all of our patients, the normal *E*/*A* was considered 1.5 (1.0-2.0). Mean values of early (*E*′) myocardial velocities were calculated to find the *E*/*E*′ ratio. Left atrial volume index (LAVI) and mean pulmonary artery (PA) pressure were also calculated. Tricuspid annular plane systolic excursion (TAPSE) was evaluated to assess right ventricular function (normal values > 20 cm/s). Moreover, 9-lead ECG was recorded and the result was analyzed by the experienced cardiologist.

### 2.4. Statistical Analysis

Data were analyzed using SPSS 24.0 (SPSS, Chicago, IL, USA). All tests were two-tailed, and *p* < 0.05 was considered to be statistically significant, as well as values < 0.1 as indicating a tendency or trending toward statistical significant. Consideration of *p* < 0.1 may be relevant, particularly in a small study [32]. Mean and standard deviation for continuous variables and percentages for categorical variables are the main descriptive statistics reported. Gaussian distribution of the data was assessed by using the Shapiro-Wilk test. Because there was no cut-off value for FABP4, patients were divided into two groups according to median serum FABP4 value to get an equal number of samples for comparison between the lower and higher groups. Discrete variables were compared with the chi-square test (Fisher exact test when appropriate) and continuous variables with Student's *t*-test (Mann–Whitney when appropriate). Pearson correlation coefficients between FABP4 and measurement parameters, including serum ferritin levels and echocardiography parameter (LAVI), were calculated.

## 3. Results

### 3.1. Patient Characteristics

Table 1 shows the characteristics of the patients with thalassemia major. The mean age was 21.9 ± 8.0 years; age range in this study was 15-53 years; 18 were male; and the mean interval of blood transfusion was 5.7 ± 7.2 weeks. The most-reported iron-chelating agent was deferasirox (43.9%). The mean serum ferritin was 4605.2 ± 3303.7, and the mean FABP4 was 21.6 ± 1.9 ng/mL.

**Figure 1:**
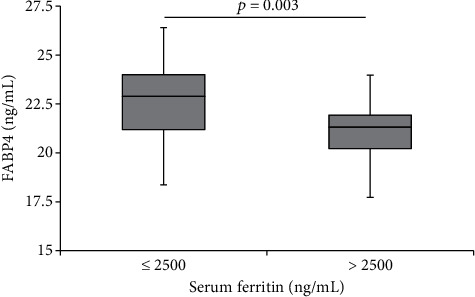
Serum fatty acid-binding protein 4 (FABP4) levels in patients with thalassemia major with and without siderosis (serum ferritin > 2500 ng/mL and ≤2500 ng/mL, respectively, *p* = 0.003).

Clinical characteristics between two groups (higher and lower groups) divided by the median serum FABP4 value (21.5 ng/mL) are compared as shown in Table 1. A tendency of difference was observed in serum ferritin levels (*p* < 0.1). There was no gender difference regarding serum FABP4 value in patients with thalassemia major. The presence of siderosis was determined using a cut-off of the serum ferritin as previously described [30]. As shown in Figure 1, patients with siderosis (serum ferritin > 2500 ng/mL) have significantly lower levels of FABP4 (*p* < 0.01).

### 3.2. Liver and Cardiac Function Stratified by FABP4

Table 2 lists the comparison results of liver and cardiac function test stratified by median serum FABP4 value. Concerning the patients with thalassemia major, there were no significant differences in terms of liver function test, including AST levels. Although not reaching statistically significant, ALT tended to decrease in the higher serum FABP4 group compared with the lower one.

Concerning cardiac function, as demonstrated in Table 2, there were significantly lower values of LVEF and LVSV in patients with thalassemia major, with higher serum FABP4 compared with those who have lower serum FABP4 (63.7 ± 5.3 vs. 67.0 ± 4.8, *p* < 0.05 and 34.6 ± 3.9 vs. 37.1 ± 3.8, *p* < 0.05, respectively). However, left ventricular posterior wall diastolic diameter (LVPWDD, 9.0 ± 2.0 vs. 8.0 ± 1.3, *p* < 0.05), LVESV (37.6 ± 12.7 vs. 29.7 ± 9.6, *p* < 0.05), *E*/*E*′ (11.1 ± 3.4 vs. 8.6 ± 1.8, *p* < 0.05), left ventricular mass index (109.2 ± 29.8 vs. 90.1 ± 17.0, *p* < 0.05), and LAVI (34.1 ± 17.1 vs. 25.8 ± 5.7, *p* < 0.05) were significantly increased in those with higher serum FABP4 levels. In terms of ECG, there were no significant differences in ECG results between the two groups. However, left ventricular hypertrophy (8.7%), conduction block (8.7), and arrhythmia (4.3%) were only seen in patients with higher serum FABP4 levels.

### 3.3. Correlation Coefficients for Serum FABP4 and Liver and Cardiac Function

**Figure 2:**
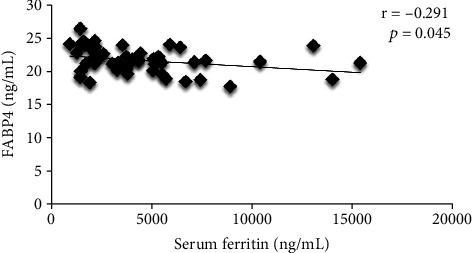
Correlation between serum ferritin and FABP4.

**Figure 3:**
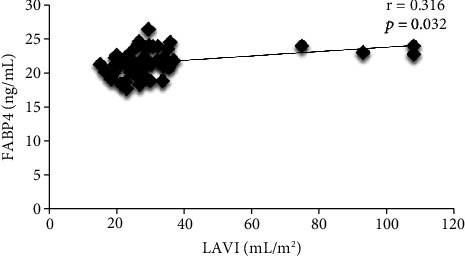
Correlation between left atrial volume index (LAVI) and FABP4.

Figure 2 shows the correlation between FABP4 and serum ferritin. There was a weak negative correlation between serum ferritin and FABP4 (*r* = −0.291, *p* < 0.05). In addition, there was a significantly moderately positive correlation between LAVI and FABP4 (*r* = 0.316, *p* < 0.05) in patients with thalassemia major, as shown in Figure 3.

## 4. Discussion

The effects of iron excess in patients with thalassemia major have remained in increasing attention because of its higher morbidity and mortality in this population. Iron accumulation may increase the production of unstable iron, which can affect reactive oxygen species production and thus perpetuate organ damage [18]. Previous work has demonstrated a favorable inflammatory profile in patients with thalassemia, especially those who developed cardiovascular diseases [33]. Additionally, iron accumulation in tissues may impact its metabolism. The role of FABP4, as one of a novel biomarker for metabolically driven low-grade and chronic inflammation [26], may provide a clue in these mechanisms that occurred in patients with thalassemia major.

Cardiovascular dysfunctions are the most serious problems in patients with thalassemia major. These complications involve fatal cardiac arrhythmias and congestive heart failure [8, 12, 14–16]. The judgment of ventricular dysfunction in patients with thalassemia major is different from nonanemic patients. Patients with thalassemia major undergo cardiovascular adjustment to chronic anemia, such as high ejection fraction, tachycardia at rest, low blood pressure, and increased end-diastolic volume [13]. Prolonged anemia also induces dyspnea that mimics signs and symptoms of cardiac deterioration. Although serum ferritin is not a sufficient surrogate for cardiac iron evaluation, noninvasive assessment may provide useful information. Mostly, diastolic dysfunction occurred earlier than systolic dysfunction [34].

There is emerging evidence that accumulation of iron may induce lipid peroxidation via oxidative stress, which results in changes of membrane fatty acid. Furthermore, these can impair metabolism and end up with an inflammatory state [35]. Additionally, a growing body of evidence indicates that FABP4 contributes to the inflammatory and metabolic pathway of cardiovascular dysfunction [22, 25, 26].

Studies in nonthalassemia patients showed that women exhibited higher FABP4 levels than men, possibly due to disparities in adipose storage or the contribution of sex hormones in the secretion regulation [36, 37]. Based on our study, the gender differences in serum FABP4 levels were absent in patients with thalassemia major. Supporting this, Vlychou and colleagues found that there was no significant difference in terms of body mass index (BMI) in male and female patients with thalassemia major [38]. Furthermore, the overall fat was lower in patients with thalassemia major compared to nonthalassemia controls [39]. Impairment both in height and in weight concurrently with lower plasma value of essential amino acids occurs in patients with thalassemia [40]. Patients with thalassemia had lower BMI in comparison to healthy controls, which was affected by chronic anemia, ferritin elevation, and noncompliance of iron chelation therapy [39]. Additionally, the conditions of chronic anemia and ineffective erythropoiesis in patients with thalassemia are related to a hypermetabolic state that result in decreased fat and deficiency in fat-soluble vitamins [13].

Left atrial enlargement appears in systolic and diastolic dysfunctions [41]. LAVI, the value of left atrial volume divided by body surface area, is recommended by the American Society of Echocardiography to measure left atrial size [42]. In this study, increased FABP4 levels are correlated with increased LAVI. It may imply that diastolic dysfunction may occur with the increased FABP4 levels. In addition, regarding the left ventricular diastolic function, the *E*/*E*′ ratio was related to increased FABP4 level. Higher FABP4 value has been associated with metabolic syndrome, inflammation, development of atherosclerosis, and insulin resistance [26, 43], and thus, various metabolic changes due to iron toxicity [5, 17, 18] may be accompanied by an increase in FABP4 level and simultaneously promote an increase in LAVI and *E*/*E*′ ratio. However, the cross-sectional design of this study prevents the demonstration of the mechanisms by which increased LAVI and *E*/*E*′ ratio are related to FABP4 levels.

This study found that increased left ventricular mass index is associated with increased serum FABP4 levels. A raised FABP4 value has been associated with diabetes mellitus, development of atherosclerosis, inflammation, and insulin resistance; and the iron accumulation in patients with thalassemia major may induce these conditions [22, 26]. Therefore, those mechanisms may simultaneously promote an increase in the left ventricular mass index. Moreover, in this study, LVEF and LVFS were lower among patients with higher FABP4 levels. In terms of range value of echocardiography parameters, it is important to use the different normal ranges for patients with thalassemia major, because in these patients without cardiac iron overload, LVEDV is increased and LVESV is decreased, which leads to increased LVSV, LVEF, and cardiac output compared to healthy nonanemic controls [4, 13]. Higher LV mass and the right ventricular indexes can be caused by a hyperdynamic state. Therefore, it is challenging to identify ventricular systolic dysfunction in patients with thalassemia major. Hyperdynamic effects related to chronic anemia facilitated to preserve myocardial fiber shortening and normal ejection fraction [44].

Interestingly, abnormalities in ECG especially left ventricular hypertrophy, conduction block, and arrhythmia were only demonstrated in patients with higher FABP4 levels. A previous study showed similar findings of ECG abnormalities in patients with thalassemia [45]. It was suggested that FABP4 may have an essential function in the regulation of cardiac depolarization and arrhythmias [27]. Additionally, patients with thalassemia major have higher chronic inflammation due to iron toxicity [19–21], which can increase the risk of cardiac arrhythmias through mechanisms involving vascular injury, myocardial fibrosis, increased sympathetic activity, and failure of ion channel [27].

Inverse correlation of serum FABP4 and serum ferritin levels in patients with thalassemia major was demonstrated in our study. This could be related to the toxicity of iron excess in adipocytes that could prevent its role to produce or secrete FABP4. Additionally, it has been described that the levels of hemoglobin and ineffective erythropoiesis were related to hypermetabolic states that affect the amounts of body fat in patients with thalassemia [13, 46]. Total cholesterol and low-density lipoprotein cholesterol were lower in patients with thalassemia than in control participants [47–49]. The levels of various nutritional biomarkers were also reduced in patients with transfusion-dependent thalassemia [50]. Although previous reports have shown the link between adiposity and FABP4, in other studies, the connection of regulation and disease correlation appears to be complex, especially in rapid loss of adiposity, which might lead to an enormous breakdown of lipid [51]. Therefore, enhanced serum FABP4 levels may still occur in condition of lower adipocyte secretion [48–51].

Higher levels of AST do not always indicate health problem because they could result from some factors such as strenuous physical exercise [9]. However, the ALT level is more specific for hepatocellular damage secondary to iron excess as the liver is the first target organ dysfunction. In this study, ALT tended to increase in patients with lower serum FABP4. This could be under the aforementioned mechanism of concomitant iron toxicity in the liver and adipocytes.

In line with previous work, this study has demonstrated a favorable inflammatory profile in patients with thalassemia, especially those who developed cardiovascular dysfunction [33]. In this study, although not classified into abnormalities, patients with thalassemia who presented reduced cardiac function based on echocardiography and ECG showed higher levels of serum FABP4. The role of FABP4, as one of a novel biomarker for metabolically driven low-grade and chronic inflammation [26], may provide a clue in these mechanisms that occurred in patients with thalassemia major. In addition, a study by Tu and colleagues showed that FABP4 is a prognostic biomarker in stroke patients [52]. Based on this study, for further investigation, it is also possible for FABP4 to be a prognostic biomarker for cardiovascular complications in patients with thalassemia because the deterioration of cardiac function in patients with thalassemia may occur progressively due to cardiac iron accumulation. This emphasized the importance of optimal iron-chelation therapy. Therefore, problems related to nonoptimal iron-chelation therapy such as noncompliance, financial problem including health insurance should be managed well.

Although this is the first study that sought to explore the correlation between FABP4 and cardiac function in patients with thalassemia major, several limitations need to be considered in the interpretation of the study findings. Data on hemoglobin typing and genetic analysis were not obtained. The height and weight (and consequently the BMI) of the patients were not obtained in this study. In addition, the parameters related to metabolism were not examined. In line with this, the measurement of inflammatory markers could potentially reveal an association between metabolic and inflammatory status in patients with thalassemia. However, estimation of iron overload was conducted using serum ferritin because of its affordability and availability; despite that, T2^∗^ magnetic resonance imaging (MRI) is the recommended method to calculate iron accumulation [53]. Single cross-sectional ferritin measurement may be misrepresentative because it may not reflect long-term ferritin levels [13]. A prospective study with a greater number of subjects and even comparison to healthy subjects is necessary for any further investigation of FABP4 in patients with thalassemia. Additionally, the inclusion of more patients with cardiac abnormalities and comparison with the gold standard of iron toxicity examination (T2^∗^ MRI) are important to make further diagnostic analysis and also adjustment of confounding factor in order to see possibility of FABP4 as a potential biomarker of cardiac iron toxicity.

## 5. Conclusions

There were correlations between serum FABP4 and cardiac function, as well as serum ferritin in patients with thalassemia major. FABP4 levels could be a potentially useful clinical biomarker for cardiac dysfunction via metabolic and inflammatory pathways due to iron accumulation and toxicity in patients with thalassemia major. Further prospective studies are needed to elucidate whether increased serum FABP4 level is a marker or a potential mechanism for iron-related cardiac involvement in patients with thalassemia major.

## Figures and Tables

**Table 1:** Characteristics in total patients and stratified by median of fatty acid-binding protein 4 (FABP4) levels (median = 21.5 ng/mL).

	Lower	Higher	Total	*p* value
Age, years (mean ± SD)	21.8 ± 7.7	22.0 ± 8.5	21.9 ± 8.0	0.929
Sex, *n* (%)				0.592
Male	9 (37.5)	9 (37.5)	18 (37.5)	
Female	15 (62.5)	15 (62.5)	30 (62.5)	
Age of first transfusion, months (mean ± SD)	54.6 ± 51.9	73.6 ± 124.1	64.1 ± 94.6	0.451
Transfusion interval, weeks (mean ± SD)	5.2 ± 2.4	6.3 ± 10.0	5.7 ± 7.2	0.187
Type of chelation therapy, *n* (%)				0.968
Deferoxamine monotherapy	2 (9.5)	2 (10.0)	4 (9.8)	
Deferiprone monotherapy	8 (38.1)	9 (45.0)	17 (41.5)	
Deferasirox monotherapy	10 (47.6)	8 (40.0)	18 (43.9)	
Deferoxamine and deferasirox	1 (4.8)	1 (5.0)	2 (4.9)	
Pretransfusion Hb, g/dL (mean ± SD)	7.3 ± 2.3	7.0 ± 1.5	7.2 ± 1.9	0.573
Serum ferritin, ng/mL (mean ± SD)	5436.8 ± 3689.9	3773.5 ± 2691.5	4605.2 ± 3303.7	0.082
FABP4, ng/mL (mean ± SD)	20.1 ± 1.2	23.1 ± 1.2	21.6 ± 1.9	0.000

Hb: hemoglobin, Student's *t*-test, Mann–Whitney, or chi-square tests were used as appropriate.

**Table 2:** Liver enzyme and cardiac function parameters stratified by median of FABP4 levels (median = 21.5 ng/mL).

	Lower	Higher	*p* value
*Liver enzymes, ng/mL (mean* ± *SD*)			
AST	64.8 ± 4.9	50.4 ± 6.3	0.114
ALT	58.8 ± 41.2	40.0 ± 22.0	0.057
*Cardiac function parameters (mean* ± *SD*)			
Echocardiography			
LVPWDD (mm)	8.0 ± 1.3	9.0 ± 2.0	0.049
LVESV (mL)	29.7 ± 9.6	37.6 ± 12.7	0.019
LVEF (%)	67.0 ± 4.8	63.7 ± 5.3	0.029
LVFS (%)	37.1 ± 3.8	34.6 ± 3.9	0.029
LV mass index	90.1 ± 17.0	109.2 ± 29.8	0.011
LV diastolic function *E*/*A* ratio (m/s)	1.6 ± 0.3	1.6 ± 0.4	0.849
LV diastolic function *E*/*E*′	8.6 ± 1.8	11.1 ± 3.4	0.003
LV diastolic function LAVI (mL/m^2^)	25.8 ± 5.7	34.1 ± 17.1	0.040
Mean PA pressure (mmHg)	17.3 ± 5.1	22.0 ± 11.0	0.081
TAPSE (mm)	22.2 ± 2.9	23.6 ± 4.2	0.206
Electrocardiography, *n* (%)			0.365
Normal	12 (52.2)	9 (39.1)	
Tachycardia	3 (13.0)	2 (8.7)	
ST-T wave changes	8 (34.8)	7 (30.4)	
Left ventricular hypertrophy	0 (0.0)	2 (8.7)	
Conduction block	0 (0.0)	2 (8.7)	
Arrhythmia	0 (0.0)	1 (4.3)	

AST: aspartate transaminase; ALT: alanine transaminase; LVPWDD: left ventricular posterior wall diastolic diameter; LVESV: left ventricular end-systolic volume; LVEF: left ventricular ejection fraction; LVFS: left ventricular fractional shortening; LV: left ventricle; LAVI: left atrial volume index; PA: pulmonary artery; TAPSE: tricuspid annular plane systolic excursion; Student's *t*-test, Mann–Whitney, or chi-square tests were used as appropriate.

## Data Availability

The data used to support the findings of this study are available from the corresponding author upon request.
